# Influence of Breast Cancer Awareness Month on Public Interest of Breast Cancer in High-Income Countries Between 2012 and 2022: Google Trends Analysis

**DOI:** 10.2196/49197

**Published:** 2024-08-12

**Authors:** Majed Ramadan, Doaa Aboalola, Sihem Aouabdi, Tariq Alghamdi, Mona Alsolami, Alaa Samkari, Rawiah Alsiary

**Affiliations:** 1 Population Health Research Section, King Abdullah International Medical Research Center King Saud Bin Abdulaziz University for Health Sciences Ministry of National Guard-Health Affairs Jeddah Saudi Arabia; 2 Department of Cellular Therapy and Cancer Research, King Abdullah International Medical Research Center King Saud Bin Abdulaziz University for Health Sciences Ministry of National Guard-Health Affairs Jeddah Saudi Arabia; 3 Department of Biology College of Science King Khalid Univerity Abha Saudi Arabia; 4 Pathology Department, King Abdulaziz Medical City King Saud Bin Abdulaziz University for Health Sciences Ministry of National Guard-Health Affairs Jeddah Saudi Arabia

**Keywords:** Google Trends, breast cancer, pandemic, awareness, public interest, cancer, cancer awareness, women, mortality rate, detection, treatment, social media, tool, education, support, internet users

## Abstract

**Background:**

Breast cancer is the most common cancer among women worldwide. High-income countries have a greater incidence and mortality rate of breast cancer than low-income countries. As a result, raising awareness about breast cancer is crucial in increasing the chances of early detection and treatment. Social media has evolved into an essential tool for Breast Cancer Awareness Month campaigns, allowing people to share their breast cancer stories and experiences while also providing a venue for education and support.

**Objective:**

The aim of this study was to assess the level of public interest in searches linked to breast cancer among a sample of high-income nations with a sizable internet user base from 2012 to 2022. We also sought to compare the proportional search volume for breast cancer during Breast Cancer Awareness Month with that during other months of the year.

**Methods:**

Google Trends was used to retrieve data on internet user search behaviors in the context of breast cancer from 2012 to 2022. Seven countries were evaluated in this study: Australia, Canada, Ireland, New Zealand, the United Kingdom, Saudi Arabia, and the United States, in addition to global data. Breast cancer relative search volume trends were analyzed annually, monthly, and weekly from 2012 to 2022. The annual percent change (APC) was calculated for each country and worldwide. Monthly and weekly data were used to identify potential trends.

**Results:**

A fluctuating pattern in APC rates was observed, with a notable increase in 2018 and a significant decrease in 2020, particularly in Saudi Arabia. Monthly analysis revealed a consistent peak in search volume during October (Breast Cancer Awareness Month) each year. Weekly trends over a 20-year period indicated significant decreases in Australia, Canada, New Zealand, and the United States, while increases were noted in Ireland. Heatmap analysis further highlighted a consistent elevation in median search volume during October across all countries.

**Conclusions:**

These findings underscore the impact of Breast Cancer Awareness Month and suggest potential influences of governmental COVID-19 pandemic control measures in 2020 on internet search behavior.

## Introduction

Breast cancer is the most common cancer among women globally, accounting for 25% of all cancer cases with an estimated 2.3 million new cases diagnosed each year [1]. In Saudi Arabia alone, there was a 5-fold increase in breast cancer incidence over 17 years [2]. Breast cancer is also the most common cancer type in the United States, with over 280,000 new cases and 43,600 deaths in 2020 [3]. The incidence of breast cancer varies among different countries and regions. High-income countries have relatively high incidence and mortality rates of breast cancer compared to low-income countries, with approximately 1 in 8 women being diagnosed with breast cancer during their lifetime [4]. A recent study on 148 countries showed that breast cancer mortality rates were lower in countries where universal health coverage for breast cancer was high [5] and the mortality rate was high in low-income countries [6]. Specifically, the number of women diagnosed with breast cancer in high-income countries is twice that in middle- and low-income countries [4]. This is partly due to better access to screening and health care, leading to earlier detection and treatment of the disease. In addition, lifestyle factors such as diet, physical activity, and alcohol consumption contribute to the incidence of breast cancer [7]. Therefore, breast cancer awareness is crucial in high-income countries given the high incidence rate, which can offer education and consequently potential for early detection and treatment [7].

A recent study in the United States showed a significant increase in public interest in breast cancer during the month of October, which is marketed as Breast Cancer Awareness Month (BCAM), from 2012 to 2021, reaching peaks in weekly relative search volume (RSV) [3]. This trend was even greater in 2020 at the beginning of the COVID-19 pandemic. Early detection of breast cancer through regular screening and self-examination can significantly improve the chances of successful treatment and survival. In particular, raising public awareness for breast cancer can decrease the breast cancer mortality rate by 20% [8]. Increased awareness of breast cancer symptoms and risk factors can also help women to identify any potential issues early on and seek appropriate medical advice [9].

BCAM is an annual campaign that takes place in October to raise awareness of breast cancer and encourage early detection and treatment. The campaign aims to educate women about the importance of self-examination, clinical examination, and mammography screening [10]. High-income countries have been at the forefront of BCAM campaigns, with various activities such as walks, runs, and fundraisers to support breast cancer research and treatment [11]. In addition, social media has become an essential tool for BCAM campaigns, allowing people to share their stories and experiences with breast cancer and providing a platform for education and support [12]. High-income countries with high rates of internet use have an advantage in using social media for breast cancer awareness campaigns, reaching a wider audience and providing more significant opportunities for engagement and participation [13]. As mentioned above, the RSV was found to be higher during BCAM, especially with respect to breast cancer donations and related events [3].

High-income countries have high rates of individuals using the internet, with an average of 89% to 95% of the population using the internet in 2021 [14]. This has implications for breast cancer awareness, education, and prevention. The internet provides access to a vast amount of information on breast cancer, including risk factors, symptoms, screening, and treatment options. In addition, social media platforms such as Facebook and Twitter provide opportunities for breast cancer awareness campaigns to reach a wider audience and engage with the public [15]. Therefore, it is essential to evaluate the public interest in breast cancer awareness using widely usable online searching websites such as Google, particularly among high-income countries with high breast cancer incidence rates [16].

Google Trends can provide valuable insights into how people are searching for and engaging with information related to breast cancer. Google Trends enables tracking and analyzing search patterns and interest in specific topics over time, providing a useful tool for researchers and health care professionals to monitor public interest and awareness [17]. This information can be used to inform targeted awareness campaigns and public health interventions, as well as to evaluate the effectiveness of existing campaigns. This type of research can also help to identify opportunities for increased awareness and education, as well as to assess the potential impact of media coverage on the public perception of breast cancer. However, there have been very few studies on the effectiveness of BCAM to improve public awareness for breast cancer [3]. Therefore, the primary aim of this study was to evaluate the public interest of breast cancer–related searches among selected high-income countries with a large number of internet users between 2012 to 2022. We further aimed to compare the breast cancer RSV during BCAM with that during other months of the year.

## Methods

### Sample and Data

We used the web-based tool Google Trends [18] to assess the level of interest in specific search queries. Our methodology adhered to the Google Trends Methodology Framework in Infodemiology and Infoveillance [19,20]. Notably, Google Trends does not disclose exact RSV figures; rather, it presents the relative number of searches within a defined region and time frame for a given query based on a scale from 0 to 100. A score of 100 signifies the zenith of query popularity, while 0 denotes minimal search activity [21]. Data from Google Trends were compiled between January 2012 and December 2022. The following 7 countries were assessed in this study: Australia, Canada, Ireland, New Zealand, the United Kingdom, Saudi Arabia, and the United States, in addition to worldwide data. The rationale for the selection of these countries was to gain a global perspective based on the trends occurring in high-income countries in which Google and YouTube are commonly used search engines [22]. Additionally, the high percentage of individuals using the internet in these high-income countries (90%-97% of the total population) facilitates analyzing the distinct trend line due to the large volume of internet and Google users in these countries [22].

### Variables

The Google Trends tool [18] was used on November 29, 2022, to retrieve data on internet user search activities in the context of breast cancer. Saudi Arabia Google Trends indicators were retrieved from January 2012 to December 2022 onward using the search terms “breast cancer” and the Arabic translation *“سرطان الثدي”.* We used both English and Arabic languages as key term search indicators. Using weekly data, yearly average Google Trends indicators were calculated for 2012 to 2022, which were used to describe the annual trend in the data.

### Ethical Considerations

We used publicly available data through Google Trends [18]. The study was approved by the Institutional Review Board of King Abdullah International Medical Research Center (SP24 J/009/03) with a waiver for informed consent as the study intended to analyze unidentified public data retrospectively. Notably, none of the queries in the Google database for this study can be associated with a particular individual. The database does not retain information about the identity, IP address, or specific physical location of any user. All research methods were performed following relevant guidelines and regulations.

### Statistical Analysis

To assess the comprehensive temporal patterns of the breast cancer RSV throughout the study period, we performed analyses on an annual, monthly, and weekly basis. Initially, we calculated the annual percent change (APC) with the 95% CI to examine the characteristics of the trend in breast cancer RSV over the specific study period for each country and worldwide, spanning 2012 to 2022. Subsequently, monthly and weekly data on breast cancer RSVs were used to discern the potential trends in terms of direction and magnitude. Considering the anticipated seasonal trend and the nonnormal distribution of the data, we used the seasonal Mann-Kendall test for the trend analysis. As outlined by Hirsch et al [23], Gilbert [24], and Helsel et al [25], the seasonal Mann-Kendall test serves the purpose of examining a monotonic trend in a variable when the data collected over time are anticipated to represent consistent changes (either upward or downward) during one or more seasons, such as months. A monotonic upward trend indicates that the variable consistently increases over time, whereas a monotonic downward trend indicates a consistent decrease over time, with the trend not necessarily being linear. The identification of seasonality suggests that the data display distinct distributions during different seasons, such as months throughout the year [26-28].

The seasonal Mann-Kendall test statistic *S*_i_ is calculated as:

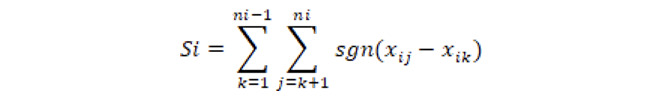



where *n* is the number of data points included in the analysis; *x_i_* and *x_j_* are the breast cancer monthly RSVs in the *i*th and *j*th month, respectively (*j*>*i*); and *sgn*(*x_j_*–*x_i_*). This function takes on the value 1, 0, or –1 according to the sign of (*x_i_*–*x_j_*) as follows:

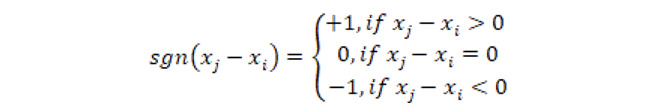



The variance is computed as:

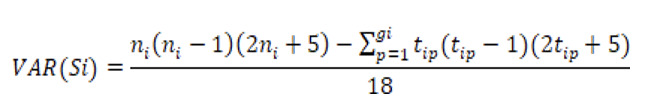



where *g_i_* is the number of tied groups for the *i*th month and *t_ip_* is the number of data points in the *p*th group for the *j*th month; *n* is the number of months included in the analysis. A tied group is a set of sample data having the same value. As *n*>10, the standard normal test statistic *Z_S_* was computed using the following formula:

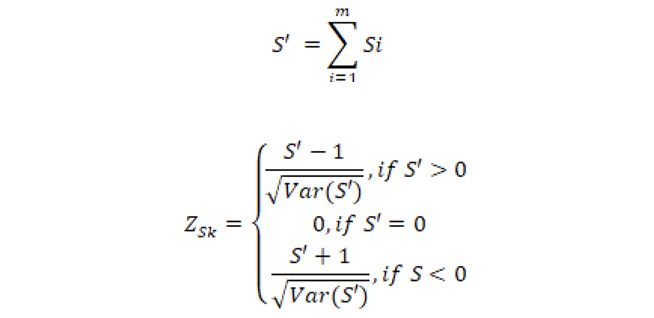



A positive value of *Z_Sk_* indicates that the data tend to increase over time, whereas a negative value indicates a decreasing trend over time [23-25].

The final step was to compare the RSV during BCAM to that of other months in the year over the study period. Toward this end, we reorganized the data by month of the year to compare between-group differences in month variables. The month variables followed neither a reliably normal nor log-normal distribution; thus, the Kruskal-Wallis test and pairwise multiple-comparisons test were used to compare the distributions of breast cancer RSVs between October (BCAM) and each other month of the year. A 2-sided *P* value <.05 was the threshold for statistical significance. All analyses were carried out in SAS 9.4.

## Results

### Trends in the APC for Breast Cancer RSVs

As shown in Table 1, from 2012 to 2022, there was substantial fluctuation in the APC for breast cancer RSVs across all countries and worldwide. In 2018, there was a significant increase in the percentage change in all countries, with the highest increase in the United Kingdom (65.9%, 95% CI 63.49%-68.32%), followed by Australia (60.58%, 95% CI 57.21%-63.94%). In 2020, there was a significant downturn in APC rates of breast cancer searches in all countries, with the highest decrease found in Saudi Arabia of –35.23% (95% CI –37.93% to –32.52%).

**Figure 1:**
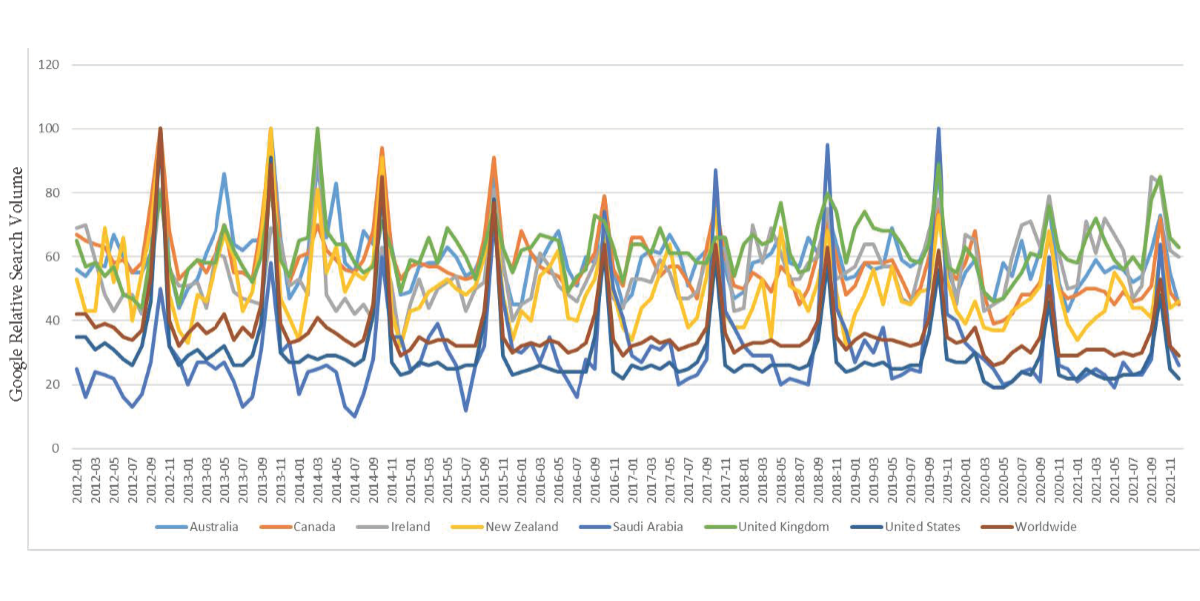
Monthly trends in breast cancer relative search volume.

Figure 1 displays the monthly breast cancer RSVs from 2012 to 2022, exhibiting a consistent uptrend peak wave in the month of October (BCAM) in each year of the study period for all included countries and worldwide.

**Table 1:** Annual percentage change rates (95% CIs) of breast cancer relative search volume (2012 to 2022).^a^

Year	Australia	Canada	Ireland	New Zealand	Saudi Arabia	United Kingdom	United States	Worldwide
2012	30.00 (55.53 to 62.26)	6.98 (4.22 to 9.74)	19.28 (16.34 to 22.21)	16.19 (14.07 to 18.31)	1.68 (–1.02 to 4.38)	5.49 (3.07 to 7.9)	12.95 (10.84 to 15.06)	7.34 (4.72 to 9.95)
2013	8.51 (5.14 to 11.87)	–5.77 (–8.53 to –3.01)	–4.54 (–7.47 to –1.6)	–2.43 (–4.54 to –0.31)	10.68 (7.97 to 13.38)	7.43 (5.01 to 9.84)	–8.84 (–10.95 to –6.73)	–5.36 (–7.97 to –2.74)
2014	0.5 (–2.86 to 3.86)	1.84 (–0.92 to 4.6)	–4.57 (–7.5 to –1.63)	1.05 (–1.06 to 3.16)	–4.47 (–7.17 to –1.76)	6.67 (4.2 to 9.08)	–4.75 (–6.8 to –2.64)	–5.00 (–7.61 to –2.38)
2015	–12.03 (–15.39 to –8.66)	–5.27 (–8.03 to –2.51	1.75 (1.18 to 4.68)	–9.02 (11.13 to –6.9)	21.7 (18.99 to 24.41)	–4.69 (–7.1 to –2.27)	–6.13 (–8.24 to –4.02)	–7.15 (–9.76 to –4.53)
2016	–0.72 (–4.08 to 2.64)	–2.72 (–5.48 to 0.03)	–0.15 (–3.08 to 2.78)	–3.05 (–5.16 to –0.93)	1.24 (–1.46 to 3.94)	–0.4 (–2.81 to 2.01)	–10.9 (–13.01 to –8.79)	–4.92 (–7.53 to –2.3)
2017	1.13 (–2.23 to 4.49)	1.13 (–1.63 to 3.89)	3.12 (0.18 to 6.05)	–1.9 (–4.01 to 0.21)	1.94 (–0.76 to 4.64)	0.26 (–2.15 to 2.67)	2.59 (0.48 to 4.7)	0.92 (–7.53 to –2.3)
2018	60.58 (57.21 to 63.94)	54.41 (51.65 to 57.17)	59.08 (56.14 to 62.01)	48.58 (46.46 to 50.69)	34.75 (32.04 to 37.45)	65.91 (63.49 to 68.32)	29 (26.89 to 31.11)	35.91 (33.29 to 38.52)
2019	2.48 (–0.88 to 5.84)	–8.58 (–11.34 to –5.82)	9.44 (6.51 to 12.3)	0.51 (–1.61 to 2.62)	0.72 (–1.98 to 3.42)	6.07 (3.6 to 8.48)	—^b^	—
2020	–8.21 (–11.57 to –4.84)	–12.02 (–14.78 to –9.26)	2.63 (–0.3 to 5.56)	–12.40 (–14.51to –10.28)	–35.23 (–37.93 to –32.52)	–15.4 (–17.81 to –12.98)	–16.46 (–18.57 to –14.35)	–13.32 (–15.93 to –10.7)
2021	0.16 (–3.21 to 3.52)	–1.00 (–3.76 to 1.76)	6.61 (3.67–9.54)	0.35 (–1.76 to 2.46)	–1.19 (–3.89 to 1.51)	12.25 (9.83 to 14.66)	1.63 (–0.48 to 3.74)	0.25 (–2.3 to 2.86)
2022	5.23 (1.86 to 8.59)	7.26 (4.5 to 10.02)	–9.54 (–12.47 to –6.61)	6.62 (4.5 to 8.73)	20.69 (17.98 to 23.39)	–0.37 (–2.78 to 2.04)	8.91 (6.8 to 11.02)	31.76 (29.14 to 34.37)

^a^Search results were normalized to the time and location of a query by the following process: each data point was divided by the total searches of the geography and time range it represents to compare relative popularity. Otherwise, places with the highest relative search volume would always be ranked the highest. The resulting numbers were then scaled on a range of 0 to 100 based on a topic’s proportion to all searches on all topics.

^b^No changes.

### Weekly Breast Cancer RSV From 2012 to 2022

**Figure 2:**
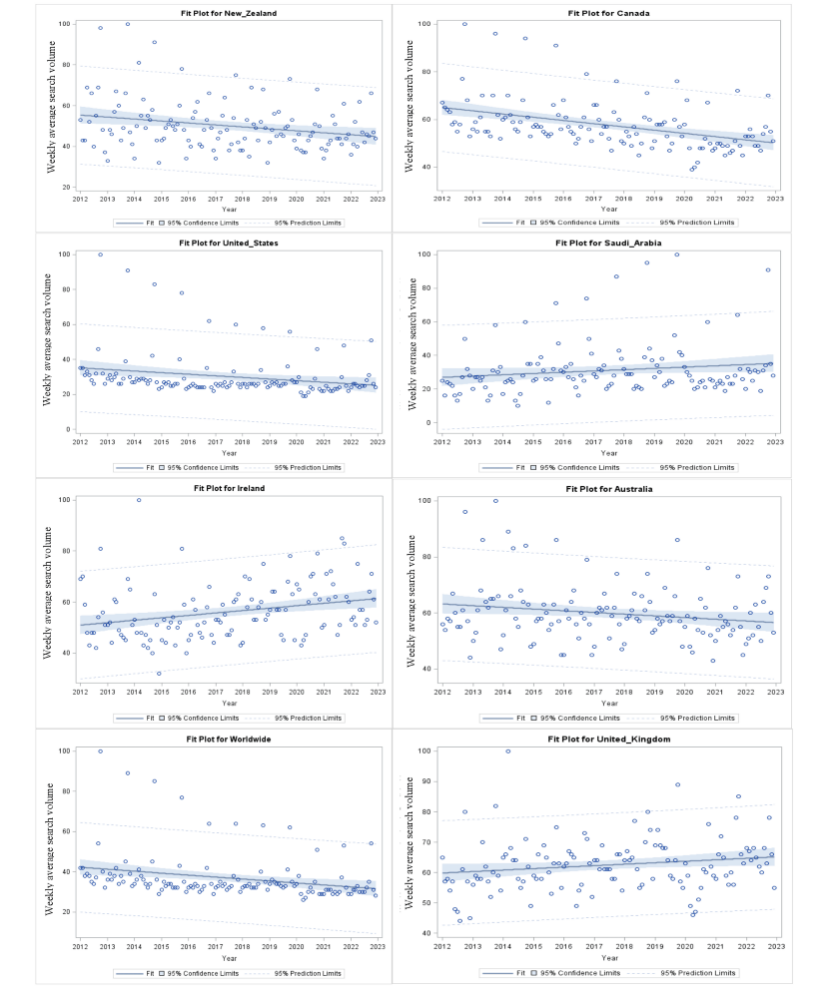
Weekly breast cancer relative search volume from 2012 to 2022. The estimated slope indicates the direction of the number of weekly new searches during the study period. The statistical significance of the magnitude and time trends calculated by the Mann-Kendall trend test are as follows: (A) *P*=.004, (B) *P*<.001, (C) *P*<.001, (D) *P*=.07, (E) *P*<.001, (F) *P*=.05, (G) *P*<.001, (H) *P*=.01.

Table 2 and Figure 2 illustrate the long-term weekly trend over 20 years. Using the Mann-Kendall trend test, 4 countries (Australia, Canada, New Zealand, and the United States) showed a statistically significant decrease in the weekly breast cancer RSV. The highest decrease was in Canada. By contrast, 2 countries showed a statistically significant increase in weekly breast cancer RSV, with the highest increase found in Ireland.

**Table 2:** Mann-Kendall trend analysis for weekly breast cancer relative search volume from 2012 to 2022.

Country	*S*^a^ (95% CI)	Mann-Kendall time trend^b^	*P* value
Australia	–0.18 (–0.34 to –0.018)	Decrease	.05
Canada	–0.42 (–0.55 to –0.27)	Decrease	<.001
Ireland	0.27 (0.11 to 0.42)	Increase	<.001
New Zealand	–0.24 (0.11 to 0.42)	Decrease	.004
Saudi Arabia	0.15 –-0.01 to 0.31)	Increase	.07
United Kingdom	0.17 (0.006 to 0.33)	Increase	.01
United States	–0.22 (–0.38 to –0.05)	Decrease	<.001
Worldwide	–0.27 (–0.42 to –0.11)	Decrease	<.001

^a^Seasonal Mann-Kendall coefficient.

^b^The Mann-Kendall trend test was applied to determine the magnitude and significance of the time trends. The estimated slope indicates the number of weekly new searches during the study period.

### Comparison of the Breast Cancer Median RSV Between Months

**Figure 3:**
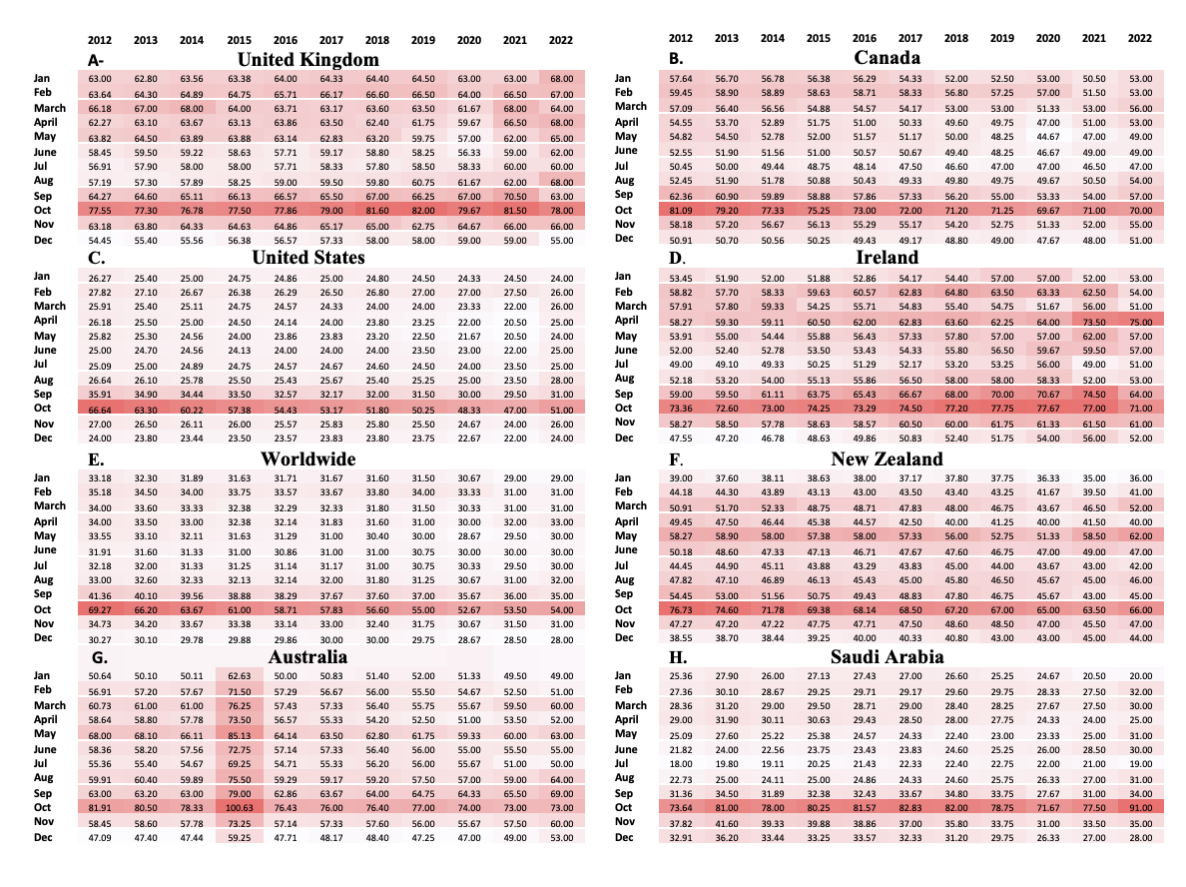
Heatmaps for median breast cancer relative search volume from 2012 to 2022 by month. The statistical significance in the trends for each country was confirmed at *P*<.001, as determined by a Kruskal-Wallis test with pairwise multiple comparisons between October and all other months. A darker color in the heatmap indicates a larger median value.

As illustrated in the heatmaps in Figure 3, there was a clear and consistent elevation in the median breast cancer RSV during the month of October every year across all included countries. The Kruskal-Wallis test, accompanied by pairwise multiple comparisons, confirmed that October exhibited a significantly higher median compared to every other month throughout the year.

## Discussion

### Principal Findings

This study demonstrated that the APCs in the breast cancer RSV on Google from 2012 to 2022 exhibited substantial fluctuations across all selected countries, with a significant drop in 2020, particularly in Saudi Arabia. Despite these changes, a constant upward peak wave in breast cancer relative searches was observed in October.

The significant fluctuations in APCs internationally and across each selected country were possibly affected by substantial global events such as the COVID-19 pandemic, which may have shifted user preferences [29]. The drop in 2020, particularly in Saudi Arabia, can be assigned to the various policy steps implemented by governments to combat the pandemic; Saudi Arabia’s rigorous policies likely deflected public interest away from breast cancer searches [30].

Internet searches for health-related topics have proven to be a very effective approach to spread knowledge of all health issues, particularly breast cancer [31].

Using Google Trends allows us to better understand the impact of BCAM on RSV, especially the trends in infoveillance, a branch of infodemiology based on observations of information-seeking behavior [32]. This will provide insight for the better management of BCAM. Previous Google Trends research demonstrated an increase in public interest and engagement in breast, colon, and oral cancer awareness campaigns, as seen by the evolving search volumes [32-34]. However, past studies have shown that BCAM regularly boosts the breast cancer RSV when compared to cancer awareness efforts for men’s cancers such as prostate cancer and testicular cancer [33,35]. In general, compared to men, women tend to seek more health-related information using electronically available tools [36-40].

Breast cancer is the most common cancer in women worldwide, including both high- and low-income countries [41]. However, based on an infographic published by the World Health Organization in 2019, breast cancer was shown to be twice as frequently diagnosed in women in high-income countries than in low- and middle-income countries [42]. Consistently, Coccia [43] demonstrated that wealthier countries have a higher incidence of breast cancer, which could be due to more efficient mammography screening [44,45]. Previous studies indicate that a lack of availability and accessibility to mammography services may account for a portion of cases going undetected, which would explain the lower incidence of breast cancer reported in some less-developed nations [45-47]. Screening and early diagnosis of breast cancer have proven to be efficient means to initiate appropriate treatment and achieve a cure for patients with cancer [48]. Therefore, we used Google Trends to track the number of searches for breast cancer in 7 high-income nations and globally between 2012 and 2022. We anticipated that the breast cancer–related RSV would be more common in high-income countries owing to the higher literacy and internet availability. Another reason could be the large public events and celebrities commissioned to increase awareness [35]. Funds allocated for BCAM marketing and the use of the pink ribbon campaign impacts the public interest in breast cancer [3]. For instance, the United States has seen higher rates of breast cancer during BCAM [45], indicating the significant impact these events have on promoting awareness, early detection, and prevention of advanced-stage disease.

The analysis of the long-term weekly trend in the breast cancer RSV from 2012 to 2022 reveals fascinating insights regarding search behaviors across countries. We discovered significant variances in search volume patterns among the studied countries. Specifically, a significant decrease in Australia, Canada, New Zealand, and the United States and a significant increase in Ireland and the United Kingdom over the 20-year period warrants further investigation into potential contributing factors such as changes in public awareness, access to health care, impact of landmark academic publications, or shifts in search engine algorithms [49,50]. Kastora et al [51] argued that analyzing geotemporal oscillations in Twitter and Google Trends for breast cancer hashtags might provide early insights into information diffusion and user involvement. These findings highlight the dynamic nature of breast cancer–related internet search activity, as well as the need for monitoring and interpreting patterns over time. More study is needed to determine the underlying causes of these observed changes and their possible implications for public health initiatives and awareness efforts. Furthermore, comparative analyses across geographies and socioeconomic circumstances may offer useful insights into the worldwide landscape of breast cancer awareness and information-seeking behaviors.

A previous study demonstrated that BCAM stimulated online searches for breast cancer [33]. Our results also confirm the importance and effectiveness of BCAM campaigns in October, as we found a correlation between the breast cancer RSV and BCAM across high-income countries and globally based on the striking seasonal increased tendency during the month of October. This implies that the awareness initiatives during this month consistently generate interest and engagement worldwide. This finding also emphasizes the importance of infoveillance to evaluate the outcome of health campaigns in general and for breast cancer in particular.

Moreover, our study revealed that over the last 10 years, the volume of searches for breast cancer varied among high-income nations that have universal health care coverage, with a significant and descending trend in the number of weekly breast cancer–related searches in Australia, Canada, New Zealand, the United States, and globally. By contrast, Ireland and the United Kingdom showed a significantly increased search volume. Despite the positive impact of BCAM in spreading awareness for breast cancer, other factors are playing a role in wealthier nations to contribute to a high breast cancer incidence, such as delayed childbearing age, obesity, smoking, hormone replacement therapy, and a higher rate of screening [44,52-54].

### Limitations

This study has several limitations that warrant consideration. First, as an ecological study, there is a risk of ecological fallacy, where trends in the specific regions we targeted might not have represented true subnational or other national trends. Second, the absence of demographic and other potential confounding factors in our analysis leaves room for the possibility of confounding bias. These unaccounted variables could influence public interest and their corresponding Google search behavior, potentially affecting the study outcomes. Third, it is acknowledged that the population seeking health information on breast cancer online may differ from the offline population. For example, not everyone searching for information on breast cancer may be connected online or use search engines, and the number of individuals connected to the internet has fluctuated over the study period (2012-2022). Therefore, this study does not precisely map the online behavior of all individuals searching for breast cancer information in the selected countries.

### Conclusion

In conclusion, the variations in the APC in the breast cancer RSV can largely be attributed to major global events such as the COVID-19 pandemic, which have the potential to shift user interest. The decrease in the APC in 2020 may be associated with variances in governmental policy measures aimed at controlling the pandemic. The uncertainty surrounding the pandemic and its impact on businesses and consumer behavior could have also contributed to the decrease in the APC. The consistent and significant peak in the breast cancer RSV during October across all countries and globally throughout the study period suggests a notable impact of BCAM on the level of public interest, as reflected by the RSV of Google Trends users. We advocate increasing the number of BCAM initiatives and spreading them throughout the year and in multiple countries to generate more awareness and reach a larger population in the countries with a downward trend. Increasing the funding toward marketing for breast cancer education will improve public awareness. This will in turn improve the screening rate and help more people eradicate the disease in its early stages.

## Abbreviations

APC: annual percent change
BCAM: Breast Cancer Awareness Month
RSV: relative search volume

## Data Availability

The data sets generated during and/or analyzed during this study are available from the corresponding author on reasonable request.
